# Pathological role of ion channels and transporters in the development and progression of triple-negative breast cancer

**DOI:** 10.1186/s12935-020-01464-9

**Published:** 2020-08-06

**Authors:** Chengli Lu, Zhiyuan Ma, Xiaoming Cheng, Huichao Wu, Biguang Tuo, Xuemei Liu, Taolang Li

**Affiliations:** 1 Department of Thyroid and Breast Surgery, Affiliated Hospital of Zunyi Medical University, Zunyi, 563003 Guizhou Province China; 2 Department of Gastroenterology, Affiliated Hospital of Zunyi Medical University, Zunyi, 563003, Guizhou Province China; 3 Digestive Disease Institute of Guizhou Province, Zunyi, Guizhou Province China

**Keywords:** Triple-negative breast cancer, Ion channels, Ion transporters, Pathological roles, Targeted therapy

## Abstract

Breast cancer is a common malignancy in women. Among breast cancer types, triple-negative breast cancer (TNBC) tends to affect younger women, is prone to axillary lymph node, lung, and bone metastases; and has a high recurrence rate. Due to a lack of classic biomarkers, the currently available treatments are surgery and chemotherapy; no targeted standard treatment options are available. Therefore, it is urgent to find a novel and effective therapeutic target. As alteration of ion channels and transporters in normal mammary cells may affect cell growth, resulting in the development and progression of TNBC, ion channels and transporters may be promising new therapeutic targets for TNBC. This review summarizes ion channels and transporters related to TNBC and may provide new tumor biomarkers and help in the development of novel targeted therapies.

## Background

Breast cancer (BC) is the common malignancy in women; its incidence is increased each year [1], and it has become a significant threat to women’s health [2]. BC is a heterogeneous disease that can be divided into multiple molecular subtypes based on estrogen receptor (ER), progesterone receptor (PR) and human epidermal growth factor receptor 2 (HER-2) expression, providing important prognostic and predictive information [3]. There are four BC subtypes depending on receptor status: luminal A, luminal B, HER-2-overexpressing and triple-negative breast cancer (TNBC). Among them, TNBC is defined as ER, PR and HER-2 negative, and it tends to affect younger women (< 40 years of age); it is prone to axillary lymph node, lung, bone metastases and has a high recurrence rate [4, 5]. Lehmann et al. classified TNBC into six subtypes based on gene cluster sequence expression: basal-like 1, basal-like 2, immunomodulatory, mesenchymal, mesenchymal stem-like and luminal androgen receptor subtypes [6]. After analyzing the RNA and DNA profile of 198 TNBC tumors, Matthew et al. classified TNBC into four subtypes, including luminal androgen receptor, mesenchymal, basal-like immune-suppressed and basal-like immune-activated subtypes [7]. The two classification methods have similarities, and both provide theoretical bases for exploring targeted therapies for TNBC.

Although TNBC is the BC subtype that responds best to chemotherapy, its recurrence and metastasis rates are higher than those of other BC subtypes [8]. Furthermore, due to the lack of classic biomarkers, TNBC lacks standard treatments guided by tumor biology, and only surgery and chemotherapy are currently available as treatments [9]. Previous studies have shown that ion channels and transporters play important regulatory roles in mammary physiology and the initiation and progression of BC [10]. However, the detailed functional role of ion channels and transporters in TNBC has not been clarified and summarized. In recent studies, upregulation of Na^+^/H^+^ exchanger 1 has been shown to promote the proliferation, migration and invasion of the TNBC cell line MDA-MB-231 [11, 12]. In addition, Ca^2+^ channels, such as mitochondrial calcium uniporter (MCU), can promote TNBC cell migration, invasion, and lung metastasis [13], and Alvarez et al. [14] reported that the two-pore domain potassium channel KCNK5 is associated with a poor prognosis in TNBC. Therefore, ion channels and transporters play important regulatory roles in the pathophysiology of TNBC, but there is currently no relevant review on this topic. Here, we review the pathological roles of ion channels and transporters, including AQPs, Cl^−^ channels, Ca^2+^ channels, K^+^ channels, and acid-base transporters, in the initiation and progression of TNBC.

## AQP channels

AQPs (which compose a family of transmembrane water channel proteins) modulate the movement of water and small solutes into and out of cells and maintain suitable concentrations of water and solutes for cell survival [15]. At least 13 AQP subtypes (AQP0-12) have been identified in mammals and are divided into two families based on transfer specificity, namely, the classic water-transporting AQP family and the solute-, water-, and glycerol-transporting glycoprotein family [16]. AQP0-2, AQP4 to AQP6-8 are mainly water-selective; AQP3, AQP7, AQP9, AQP10 and AQP12 also transport glycerol and possibly other small solutes. AQPs also play roles in the transport of ammonia, urea, carbon dioxide, metalloids, nitric oxide and certain ions [17]. Expression of AQP1, AQP3-5 and AQP10-12 has been detected in normal human mammary tissue and is closely related to milk secretion [18, 19]. In addition, deletion of “CCAAT”/enhancer binding protein (a family of transcription factors) β isoforms results in changes in mammary ductal morphogenesis and changes in expression of transport proteins such as AQP5, suggesting that AQP5 may be involved in mammary development [20]. Recent studies have shown that AQPs play carcinogenic roles by promoting angiogenesis, enhancing invasive and metastatic potential, and enhancing the transport of reactive oxygen species (ROS) [21, 22]. In female-specific cancers, such as BC, AQP1, 3, and 5 are the most important AQPs, and they are been reported to be upregulated [23].

AQP1, the membrane protein, was the first reported mammalian AQP and plays a significant role in tumor cell migration, proliferation and angiogenesis [24]. Clinical studies have shown that patients with TNBC have higher levels of AQP1 expression and that upregulation correlates with a poor prognosis [25, 26]. AQP1 expression is induced by hypoxia through the E-Box/ChoRE transcription element, which is affected by increased glucose consumption and metabolism [27]. AQP1 expression has been detected only in a subgroup of CK14-positive basal-like breast cancer (BLBC) cases [25]. CK14 has been used as a marker of basal mammary epithelial cells with *in vivo* regenerative ability in studies on mammary gland progenitor and stem cells [28]. Therefore, it is speculated that expression of AQP1 is related to the stem cell characteristics of BLBC cells. Hu et al. demonstrated that AQP1 upregulation promotes extravasation and increases migration in vivo and in vitro in the mouse TNBC cell line 4T1, suggesting that this aquaporin enhances the rate of cell migration by promoting water permeability in cell protrusions [29]. Thus, upregulation of AQP1 can promote the proliferation, migration and invasion in TNBC cells. Moreover, in vivo experiments have shown that AQP1 deficiency can reduce tumor mass, volume, vessel density and lung metastases in MMTV-PyVT (mouse mammary tumor virus-driven polyoma virus middle T oncogene) mice, and inhibition of AQP1 function and/or expression is predicted to attenuate angiogenesis via reduced migration and invasion of endothelial cells [30]. Recently, Irene Abreu-Rodriguez et al. [31] revealed that AQP1 expression is also responsive to hypoxia-inducible factor (HIF), which may play a role in the VEGF-independent signaling mechanism inducing angiogenesis in a hypoxic environment. Helen et al. [32] also reported that the triterpenoid saponins bacopaside I and bacopaside II can synergistically reduce the transcriptional expression of AQP1, and inhibit proliferation, migration and invasion in MDA-MB-231 cells. Similarly, ginsenoside Rg3, a compound with anticancer activity isolated from ginseng, inhibits AQP1 to attenuate cell proliferation through a mechanism that involves downregulation of AQP1 to induce cell cycle arrest in G0/G1 phase by inhibiting cyclin D and E and inhibition of chemoattractant-induced cell migration and invasion by blocking AQP1-mediated water flux in MDA-MB-231 cells [33]. These findings indicate that AQP1 plays an important role in the development and progression of TNBC.

Overexpression of AQP3 has been detected in the membranes and cytoplasm of TNBC tumor cells and is significantly associated with poor prognosis [34]. Xu-Chen Cao et al. [35] found that the presence of fibroblast growth factor-2 (FGF-2) induced cell migration and metastasis in MDA-MB-231 cells by increasing AQP3 expression. Moreover, FGF receptor kinase (FGFRK) inhibitors, PI3K inhibitors, and MEK1/2 inhibitors all inhibit AQP3 expression, suggesting that FGF receptor kinases increase AQP3 expression and promote FGF-2-induced cell migration by initiating downstream PI3K and ERK pathways. In addition, CuSO_4_, a water transport inhibitor of AQP3, inhibits migration in MDA-MB-231 cells; AQP3 downregulation reduces the proliferation, invasion and migration of MDA-MB-231 cells while increasing sensitivity to 5-fluorouracil chemotherapy. The mechanism may be related to a decrease in glycerol permeability caused by AQP3 downregulation [36]. Overall, these findings demonstrate that AQP3 plays a pivotal role in the initiation and progression of TNBC, and specific inhibitors of AQP3 in clinical applications may improve the therapeutic. effect of TNBC patients.

Similarly, overexpression of AQP5 in the membrane and cytoplasm of TNBC cells has been detected and is significantly associated with poor prognosis [34]. Moreover, patients with higher Ki-67 expression are more likely to have abnormal AQP5 protein expression than patients with lower Ki-67 expression [34]. Ki-67 is a widely accepted proliferation marker [37, 38], and it is speculated that upregulation of AQP5 may promote proliferation in TNBC cells.

In summary, AQP1, AQP3, and AQP5 are significantly upregulated in TNBC; this upregulation is related to a poor prognosis and can promote the proliferation, migration and invasion of TNBC cells. These AQPs are promising new targets for the diagnosis and treatment of TNBC.

## **Cl**^**−**^**channels**

### CFTR

CFTR is a member of the ATP-binding cassette transporter family that localizes at the apical membranes of normal epithelial cells. CFTR is mainly responsible for conducting HCO_3_^−^ and Cl^−^ and promoting HCO_3_^−^ secretion in many tissues, including the airway, intestines and pancreas [39]. However, when the extracellular concentration of Cl^−^ is higher than 40 mmol/L, the permeability of CFTR to Cl^−^ is much greater than that of CFTR to HCO_3_^−^; thus, CFTR mainly conducts Cl^−^ under physiological conditions [40]. CFTR can also transport two other anions, glutathione and thiocyanate, which are involved in airway inflammation and oxidative stress [41, 42]. Interestingly, Pierre et al. [43] reported that CFTR is required for the tightly connected functions of normal epithelial tissues; loss of CFTR reduces epithelial resistance and epithelial integrity, and this effect is not related to the anion channel function of CFTR.

CFTR has been reported to be associated with several cancers, such as cervical cancer [44], colorectal cancer [45], prostate cancer [46] and BC [47]. Significant downregulation of CFTR expression is observed in BC tissue compared to normal mammary tissue [48]. Zhang et al. demonstrated that overexpression of CFTR inhibits EMT, invasion and migration in MDA-MB-231 cells via a mechanism that involves CFTR inhibition of NFκB targeting of urokinase-type plasminogen activator [47]. In addition, CFTR overexpression inhibits the EMT and the invasiveness of MDA-MB-231 cells and reduces lung metastasis in xenograft models. Increasing evidence reveals that downregulation of CFTR occurs after treatment with EMT-inducing factors such as TGF-β, suggesting that as a downstream effector, CFTR plays important roles in mediating various EMT effects [49, 50]. Moreover, hypermethylation of the cancer genome leads to activation of oncogenes or suppression of tumor-suppressor genes, thereby resulting in tumorigenesis [51]. It has also been observed that the methylation level of CFTR in BC tissues is much higher than that in normal tissues, and treatment with DNA methylation inhibitors in TNBC cell lines (MDA-MB-231 and MDA-MB-435) can rescue CFTR mRNA, indicating that CFTR methylation plays an important role in TNBC [52]. ΔF508 is the most common mutation in CFTR, causing the protein to be retained and degraded in the endoplasmic reticulum due to misfolding [53]. It is worth noting that although there is no difference in the incidence of BC between ΔF508 carriers and noncarriers, patients with ΔF508 CFTR mutations all have grade III cancer, indicating that CFTR defects are associated with BC progression [54]. Therefore, CFTR methylation or mutation need to be further investigated in the future, which may provide novel therapeutic intervention for TNBC.

### Chloride channel 3

The chloride channel (ClC) family also plays an indispensable role in the transport of Cl^−^ [55]. There are nine family members in humans, which are divided into two categories based on their distribution and physiological function: (1) Cl^−^ channel proteins (ClC1, ClC2, ClCKa and ClCKb), which mainly exist in the plasma membrane and play roles in stabilizing membrane electric potential or mediating epithelial transport; and (2) Cl^−^/H^+^ reverse transporter proteins (ClC3-7), which mainly exist in the vascular intima of the endosome-lysosomal pathway and are localized at the plasma membrane only to a limited extent due to protein degradation and hydrolase activity [56, 57]. In recent years, it has been discovered that ClC3 can transport one hydrogen ion in exchange for two chloride ions [58], with important roles in cancers such as nasopharyngeal carcinoma [59] and BC.

ClC3 overexpression is observed in tissues and the TNBC cell line MDA-MB-231 [60, 61]. Studies by Zhou et al. revealed that knockdown of ClC3 downregulates expression of cyclin D1 and cyclin E and increases levels of p21, indicating that knockdown of ClC3 can block the cell cycle of MDA-MB-231 cells at G0/G1 phase, inhibiting cell proliferation. Moreover, knockdown of ClC3 suppresses tumor growth in xenograft models and significantly reduces levels of pERK1/2 in MDA-MB-231 cells. This indicates that ClC3 can promote the progression of TNBC by acting on the ERK1/2 signaling pathway [60]. Nevertheless, relative research on ClC3 in TNBC is still very limited, and extensive work is needed in the further.

## Ca^2+^ channels

Ca^2+^ is a key nutrient in milk that plays a vital role in the mineralization of bones and teeth, and as a second messenger, ionized Ca^2+^ is a key regulator of proliferation, migration, cell cycle progression and apoptosis [62]. The level of Ca^2+^ is very low in the cytoplasm (~ 10^− 7^ mol/L), whereas it is somewhat higher in organelles (~ 10^− 5^ mol/L) and highest in the extracellular level milieu (~ 10^− 3^ mol/L). Hence, a small amount of Ca^2+^ can significantly change intracellular levels to activate downstream signaling molecules, including calmodulin, nuclear factor of activated T-cells (NFAT), NFκB, calmodulin-dependent protein kinase II, calpain and others [63, 64]. In nonexcitatory mammary cells, calcium channels play important roles in lactation and the maintenance of normal physiological functions [65, 66].

Continuous increases in intracellular Ca^2+^ levels will drive expression of oncogenes, resulting in tumor growth and development, especially the metastatic behavior of cancer cells, and conferring tumor cells with resistance to apoptosis [67]. Abnormal expression of several Ca^2+^ transporters and ion channels, such as calcium release-activated calcium modulator 1 (Orai1), has been observed in TNBC and may lead to oncogenic Ca^2+^ signaling [68]. Interestingly, specific changes in the expression and function of Ca^2+^ channels are related to hormone receptor status and differ significantly among BC subtypes [69].

### Calcium modulator 1

Ca^2+^ influx mainly depends on store-operated calcium channels (SOCCs). When the Ca^2+^ concentration in the endoplasmic reticulum declines to a certain level, the STIM (stromal interaction molecule), which is located on the endoplasmic reticulum membrane, moves to a position close to the highly selective calcium channel protein Orai on the cell membrane. Subsequently, Orai is activated to cause Ca^2+^ influx, and store-operated calcium entry (SOCE) is initiated, thereby replenishing the calcium store. Some researchers have proposed that the canonical transient receptor potential (TRPC) also participates in the above process, though the mechanism remains controversial. There are two different claims: that both Orai and TRPC form independent channels activated by the STIM protein and that Orai and TRPC subunits form heterochannels triggered by STIM [70]. There are three Orai1 isomers (Orai1 to Orai3) and two STIM homologs (STIM1 and STIM2). SOCE has been found to be primarily mediated by Orai1 and STIM1 in TNBC [71]. Compared with that in nonmalignant breast epithelial cells, expression of Orai1 and STIM1 is significantly higher in TNBC cell lines and is associated with a poor prognosis [72, 73]. Liu et al. [74] reported that hypoxia can induce expression of Orai1, Notch1 and Jagged-1, and Orai1 is significantly downregulated after blockade of Notch signaling, suggesting that hypoxia can increase Orai1 expression in TNBC by activating Notch signaling (Notch1/Orai1/SOCE/NFAT4 axis). Similarly, Mognol et al. [75] found that Orai1 promotes the invasion and angiogenesis of TNBC cell lines and activates NFAT4, which can regulate genes involved in the cell cycle, apoptosis, angiogenesis and metastasis. In addition, Yang et al. [76] demonstrated that Orai1 and STIM1 promote the migration and invasion of MDA-MB-231 cells both in vivo and in vitro, and the authors proposed that these proteins may at least partially control cell migration by regulating focal adhesion turnover. Furthermore, treatment with TGF-β can reduce expression of STIM1, whereas blockade of SOCE can impair TGF-β-induced G0/G1 cell cycle arrest and inhibit the proliferation of MDA-MB-231 cells [77]. Based on the above research, Orai1 and STIM1 may be new therapeutic targets for TNBC. Indeed, some selective SOCE inhibitors have shown encouraging inhibitory effects on TNBC, but they are still only in the preclinical trial stage. For example, phemindole, a di-indole derivative, reduces SOCE by downregulating STIM1 expression, significantly inhibits the proliferation and migration of MDA-MB-231 cells, reduces the growth of solid tumors in mouse models and produces a targeted antitumor effect in TNBC [78]. In addition, Miroslava Didiasova et al. [79] revealed that elevated cell surface-associated enolase-1 (ENO-1) expression correlates with augmented MDA-MB-231 cell migratory and invasive properties. Pharmacological blockade (a selective SOCC inhibitor, NS1643) or knockdown of STIM1 or Orai1 reduces ENO-1-dependent migration of MDA-MB-231 cells. These results demonstrate the pivotal role of SOCE in the regulation of ENO-1 exteriorization and thus in the modulation of TNBC cell migratory and invasive properties, indicated that Orai1 and STIM1 might be promising threptic targets for TNBC.

### Secretory pathway Ca^2+^-ATPase

The secretory pathway Ca^2+^-ATPase (SPCA) can direct Ca^2+^ and Mn^2+^ from the cytoplasm to the Golgi and post-Golgi vesicles. Two isotypes (SPCA1 and SPCA2) are known, and the distribution and function of the two differ. SPCA1 is commonly expressed in mammalian tissues; expression of SPCA2 is limited to highly absorptive and secretory epithelial cells, including mammary and salivary gland cells [80]. SPCA1 is highly expressed in TNBC cell lines, and SPCA2 is highly expressed in cell lines of other subtypes [81]. Interestingly, based on clinical samples, Desma et al. reported SPCA1 levels to be significantly elevated in the basal subtype of BC compared with all other subtypes, and it is worth noting that changes in its expression affect posttranslational modification and transport of certain proteins important for tumor progression without significantly changing cytosolic calcium signaling; SPCA1 inhibition also decreased MDA-MB-231 cell proliferation [82]. Moreover, SPCA1 is a key regulator of insulin-like growth factor receptor (IGF1R) processing in TNBC cells and promotes the production of functional IGF1R; IGF1R activity is associated with poor prognosis, suggesting that targeting SPCA1 is an alternative IGF1R-inhibiting strategy [82, 83]. Overall, upregulation of SPCA1 may promote the initiation and progression of TNBC. The main mechanism reported to date involves SPCA1-mediated increase in functional IGF1R expression.

### Mitochondrial calcium uniporter

Upregulation of MCU expression on the mitochondrial membrane is closely related to a poor prognosis in BC [84]. miR-340 correlates negatively with the metastatic potential of TNBC cells [85]; it may directly inhibit MCU expression to reduce glycolysis and exercise capacity, and knockdown or inhibition of MCU inhibits the growth, invasion and metastasis of MDA-MB-231 cells [13]. Interestingly, Anna et al. [86] demonstrated that mitochondrial Ca^2+^ uptake is required for TNBC progression in vivo and that absorption of Ca^2+^ by mitochondria promotes the production of sustained mitochondrial reactive oxygen species, activating the HIF-1α signaling pathway and promoting tumor growth and metastasis. In addition, inhibiting or silencing MCU also block serum-induced migration of MDA-MB-231 cells and reduce serum or thapsigargin-induced SOCE, suggesting that MCU promotes TNBC cell migration by regulating SOCE [87]. The above results indicate that overexpression of MCU may play an important oncogenic role in the growth, invasion and metastasis of TNBC cells. However, the precise mechanism is unclear.

Other promising calcium channel targets in TNBC include TRPV6 [88]. Overall, calcium channels are promising targets for TNBC treatment, but most compounds targeting these channels are only in the preclinical trial stage. Thus, further research is needed.

## K^+^ channels

Through the action of Na^+^/K^+^-ATPase, two K^+^ molecules are transported into a cell in exchange for three sodium molecules, which increases the intracellular K^+^ concentration. K^+^ channels on the cell membrane are numerous, and in humans, more than 90 genes encode major K^+^ channel subunits [89]. K^+^ channels play key roles in maintaining acid-base balance by functioning in concert with the Na^+^/H^+^ exchanger and Na^+^/K^+^-ATPase [90], controlling electrical excitability of nerves and muscles, and participating in energy metabolism and other physiological processes. In addition, K^+^ channels can help regulate cell proliferation and cell cycle progression and are involved in tumorigenesis [91]. Many studies have reported dysregulated K^+^ channel expression in human cancers, including BC, astrocytic-type brain cancer and prostate cancer [92, 93]. Tumor-related K^+^ channels can be divided into four main categories according to their domain structures and activation mechanisms: (1) voltage-gated potassium channels, which are controlled by changes in membrane potential; (2) calcium-activated potassium channels, which are activated by intracellular calcium; (3) inwardly rectifying potassium channels; and (4) two-pore-domain potassium channels (K2P, KCNK) [94]. However, the carcinogenic mechanism of K^+^ channels remains rather clear. Nuria et al. [95] proposed that K^+^ channels may participate in and regulate tumor progression through permeation-related mechanisms (including changes in membrane potential, Ca^2+^ driving forces and cell volume regulation) and nonconductive mechanisms (dependent on protein-protein interactions).

The Kv11.1 channel (also known as human ether-a-go-go-related gene 1, hERG1) is not expressed in normal breast cells but is expressed in BC, with a relationship with subtype. Indeed, TNBC exhibits lower expression of Kv11.1 compared with other subtypes [96]. Olivia Crociani et al. [97] showed that the mRNA levels of Kv11.1 change throughout the cell cycle, peaking in G0/G1 phase. Moreover, Lansu et al. [98] reported that stimulation of Kv11.1 led to inhibition of proliferation in MDA-MB-231 cells and that an agonist, the diphenylurea derivative NS1643, caused a significant inhibition of cell proliferation. This phenomenon can be linked to a rapid decrease in the cyclin E2 protein level, which causes accumulation of cells in G0/G1 phase and an increase in tumor suppressor proteins and markers for cellular senescence, including p21, p16^INK4a^ and β-galactosidase activity. Therefore, Kv11.1 inhibits TNBC cell proliferation by activating a cellular senescence program [98]. Breuer et al. confirmed that NS1643 reprograms the EMT by attenuating the Wnt/β-catenin signaling pathway, inhibits cancer cell stemness, and significantly reduces the metastatic spread of breast tumors in a MDA-MB-231 mouse model [99]. Regardless, cardiotoxicity is an important limiting factor for potential therapeutic molecules acting on Kv11.1. Although the activator is well tolerated in BC, potential effects include tachycardia [100]. Overall, the potential benefits of Kv11.1 activators as anticancer drugs outweigh their side effects.

In addition, many other channels are altered in TNBC. For example, some K2P channels with differential expression may serve as novel molecular markers associated with TNBC. RNA-Seq analysis of K2P channels has shown that overexpression of KCNK5, KCNK9, and KCNK12 and low expression of KCNK6 and KCNK15 are related to TNBC [101]. The above findings indicate that K^+^ channels play an important role in TNBC and are expected to be diagnosis markers.

## Acid-base transporters

The pH of milk is significantly lower than that of plasma, indicating that there may be some acid-base transporters in the mammary gland that regulate the pH between the extracellular fluid and milk [102]. A uniform feature among solid tumors with high metabolic and proliferative rates is a significantly different pH from that of normal tissue [103]. Cancer cells can maintain a weakly acidic intracellular pH that is even more alkaline than that of normal cells, suggesting that tumor cells have a powerful pH regulation system [104].

The Na^+^/H^+^ exchanger (NHE), a membrane transporter, mainly catalyzes the exchange of intracellular H^+^ for extracellular Na^+^ in mammals, thereby maintaining the pH balance inside and outside the cell [105, 106]. There are 10 subtypes of NHE, with tissue- and membrane-specific expression patterns. NHE1-5 are located on the plasma membrane, and NHE6-9 are on intracellular organelle membranes; NHE10 is only expressed in osteoclasts [107]. In addition, NHE plays indispensable roles in maintaining normal mammary structure and physiological functions [108]. NHE1 (SLC9A1) is universally expressed in epithelial cells and upregulated in BC tissues compared to normal tissues [109]. Studies have shown that hypoxia, various growth factors, and hormones, among others, can activate NHE1, and enhanced NHE1 activity can reduce extracellular pH and promote metastasis of MDA-MB-231 cells [110]. Furthermore, it has been proposed that NHE1 promotes metastasis and remodeling of the extracellular matrix by acidifying the extracellular microenvironment [111]. In addition, NHE1 knockdown reduces the migration, invasion, and growth of xenograft tumors of MDA-MB-231 cells, increasing the susceptibility of these cells to paclitaxel [112, 113]. Moreover, knockdown of NHE1 or NBCn1 (SLC4A7) in the MDA-MB-231 cell line significantly reduced the steady-state intracellular pH value after acid load, the ability to restore pHi and the primary tumor growth of xenografts in vivo, but NBCn1 knockdown prolonged tumor-free survival and reduced cell proliferation [114, 115]. It has been confirmed that NHE1 and NBCn1 promote the development of TNBC through different mechanisms. There are two main NHE1 inhibitors, amiloride and cariporide, which are more effective than amiloride and highly selective [116]. Amiloride is a potassium-sparing diuretic and has blocking effects on a variety of ion channels, such as NHE and the Na^+^/Ca^2+^ exchanger. Cariporide is a highly specific and powerful NHE1 inhibitor that is relatively well tolerated in humans with heart disease [117]. Moreover, a study has suggested that KR-33028, a novel small molecule inhibitor of NHE1, produces a cellular phenotype comparable to that of NHE1 knockout cells and significantly decreases rates of migration, invasion and colony growth in TNBC cell lines MDA-MB-231, MDA-MB-468 and Hs578T [118]. The above findings suggest that NHE1 may play an important role in the progression TNBC.

Additionally, other acid-base transporters are also altered in TNBC and are expected to emerge as new targets for TNBC treatment. For instance, NBCe1 (SLC4A4) knockdown reduces cell proliferation, invasion and migration in TNBC cells expressing high levels of NBCe1 [119]. The above findings all suggest that the acid-base transporters have essential functions in the occurrence and development of TNBC, but further research is needed.

## Conclusions

**Fig. 1:**
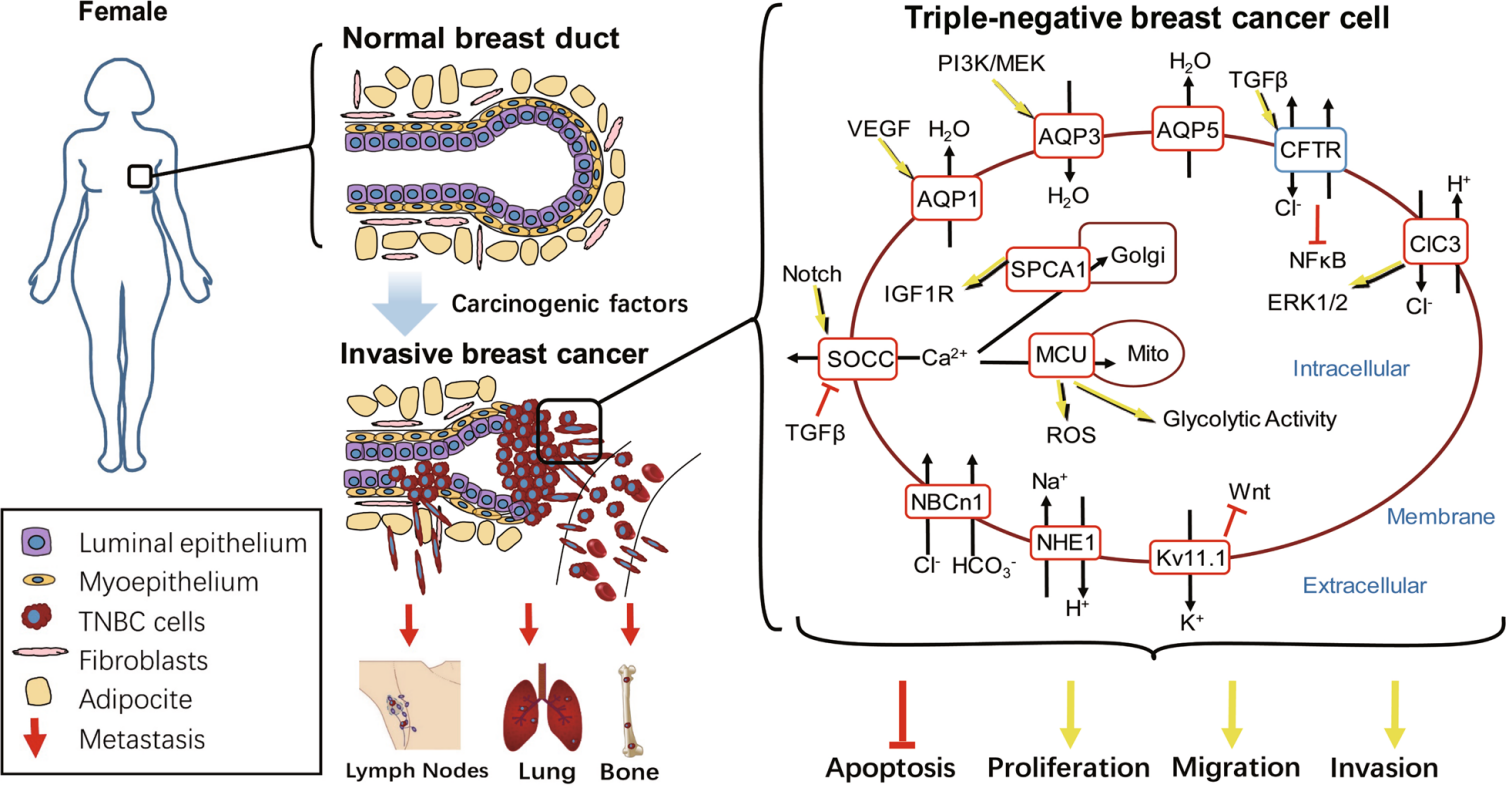
Pathological roles of ion channels and transporters in triple-negative breast cancer cells. Alteration and dysfunction of AQPs, Cl^−^ channels, Ca^2+^ channels, K^+^ channels, Na^+^/HCO_3_^−^ transporter and Na^+^/H^+^ exchanger results in abnormality of ion transport and disorder of multiple signaling pathway, including WNT, PI3K, TGFβ, Notch and VEGF, etc., eventually promoting TNBC cell proliferation, migration and invasion, but inhibiting apoptosis. (The red boxes indicate upregulation, the blue box indicate downregulation, the black arrows indicate the directions of ion transport, and the yellow arrows indicate promotion.)

Dysfunction of ion channels and transporters in the mammary resulted in development and progression of TNBC. Despite extensive work has been performed to investigate the expression pattern, functional diversity, regulatory mechanism and pathophysiology of different ion transporters in TNBC, the systematic review is rare in this field. Therefore, this review focuses on different pathological function of multiple families in the development and progression of TBNC, including the AQPs, Cl^−^ channels, Ca^2+^ channels, K^+^ channels and acid-base transporters (Fig. 1; Table 1). We hope that we can provide a basic, systemics and summarised knowledge to this field, advocating researchers play more attention on the pathophysiological role of ion channels and transporters in the development and progression of TNBC, which may provide novel targets for the clinical diagnosis and treatment of TNBC.

**Table 1:** Summaries of various of ion channels and transporters antagonists experiment in the triple-negative breast cancer

Ion channels and transporters	Transported ions	Alteration in TNBC	Roles in TNBC	Targeted TNBC cell lines	Antagonists
AQP1	Water	Upregulation	Promote proliferation, migration, invasion and induce angiogenesis	CK14 (+) BLBC, 4T1 and MDA-MB-231	Bacopaside I and bacopaside II [32], Ginsenoside Rg3 [33]
AQP3	Water and small solutes	Upregulation	Promote proliferation, invasion migration and increase sensitivity to 5-fluorouracil chemotherapy	MDA-MB-231	CuSO_4_ [35]
Calcium modulator 1	Ca^2+^	Upregulation	Promote proliferation, migration, invasion and angiogenesis	MDA-MB-231	Phemindole [78]
NHE1 (SLC9A1)	Na^+^and H^+^	Upregulation	Promote proliferation, migration, invasion and growth, reduce sensitivity to paclitaxel	MDA-MB-231, MDA-MB-468 and Hs578T	Amiloride and cariporide [116], KR-33,028 [118]

## Data Availability

Not applicable.

## Abbreviations

AQP: Aquaporin
BLBC: Basal-like breast cancer
BC: Breast cancer
CFTR: Cystic fibrosis transmembrane conductance regulator
ClC: Chloride channel
ENO-1: Enolase-1
ER: Estrogen receptor
EMT: Epithelial-mesenchymal transition
FGF-2: Fibroblast growth factor-2
FGFRK: FGF receptor kinase
HER-2: Human epidermal growth factor receptor 2
hERG1: Human Ether-a-go-go-related gene 1
HIF: Hypoxia-inducible factor
IGF1R: Insulin-like growth factor receptor
K2P (KCNK): Two-pore-domain potassium channels
MCU: Mitochondrial calcium uniporter
MMTV-PyVT: Mouse mammary tumor virus-driven polyoma virus middle T oncogene
NFAT: Nuclear factor of activated T-cells
NHE: Na^+^/H^+^ exchanger
Orai1: Calcium release-activated calcium modulator 1
PR: Progesterone receptor
ROS: Reactive oxygen species
SLC: Solute carrier
SOCE: Store-operated calcium entry
SOCCs: Store-operated calcium channels
SPCA: Secretory pathway Ca^2+^-ATPase
STIM: Stromal interaction molecule
TNBC: Triple-negative breast cancer
TRPC: Canonical transient receptor potential
